# 

**Published:** 1881-07-20

**Authors:** 


### TABLE OF CONTENTS.

S.G.O. INDEXED

ORIGINAL AND SELECTED ARTICLES.

Conversations upon the Physical and Mental Hygiene of Girlhood, by T. S. Powell, M.D., Atlanta, 241. Duration of Pregnancy, 249. Our Reply, 250 Rotheln, by A. B. Loving, M.D., of Arkansas, 251. Epidemic Metastatic Parotiditis, by C. H. Miller, M.D., of Kansas, 252. Prevention of Scarlet Fever, by J B. Garrison, M.D., of Ark., 253. Episiotomy in Dystocia, by F. O. Nagle, M.D., of Pa., 255. Catarrh and Dyspepsia, by A. P. Whitehead, M.D., 257.

ABSTRACTS AND GLEANINGS.

Nature of Green Vomit, 258. Remedies for Headache, 259. Cerebro-Spinal Meningitis, 260. Retained Placenta, 262. Important Modification of the Ordinary Anæsthetic Method, 263. Sunstroke and its Treatment, 264. Interval Between Apparent and Real Death in Asphyxia from Lack of Air, 265. Action of Benzoic Acid, 266. National Board of Health and International Sanitary Conference of 1881; Earache; Cancer of the Tongue, 267. Gastric Remittent Fever; Thapsia Plaster; Lemon Juice in Diphtheria, 268. Benzoate of Sodium in Acute Rheumatism; How to Vaccinate; Old Treatment of Dysentery Revived, 269. Chloroform in Diphtheria; Propagation of Syphilis by Razor; Death Smell, 270.

SCIENTIFIC ITEMS.

How Long Man May Live; The Process of Rumination, 271. Value of the Dentaphone, 272.

PRACTICAL NOTES AND FORMULÆ.

Neuralgia and Rheumatism; Salicylates in Serous Diarrhœa; Summer Diarrhœa of Children, 273. Summer Dysentery and Diarrhœa of Teething Children; Impotency—Nocturnal Emissions; Iodide of Potassium in Tyhoid Fever; Peppermint in Neuralgia and Rheumatism; Constipation, 274. Emulsions of Cod-Liver Oil; Ergotine in Prolapsus Ani; Cystitis; Amenorrhœa, 275. Treatment of Diphtheria; Viburnum Opulus; Venereal Warts, 276.

EDITORIAL AND MISCELLANEOUS.

Editorial Notices; Brilliant Reception, 277. Forgetfulness, etc.; Association of Medical Editors, 278. American Medical Association; Medical College Association, 279. Special Notices, 240.

